# Prevalence and predictors of under-nutrition among school children in a rural South-eastern Nigerian community: a cross sectional study

**DOI:** 10.1186/s12889-018-5479-5

**Published:** 2018-05-02

**Authors:** Rufina N. B. Ayogu, Ifeoma C. Afiaenyi, Edith U. Madukwe, Elizabeth A. Udenta

**Affiliations:** Department of Nutrition and Dietetics, University of Nigeria, Nsukka, Nsukka, Nigeria

**Keywords:** Stunting, Underweight, Thinness, Vitamin A deficiency, Zinc deficiency, Rural, School children, Determinants, Nigeria

## Abstract

**Background:**

School children in developing countries like Nigeria are faced with numerous nutrition and health problems. Lack of functional school health and nutrition programmes in Enugu state, Nigeria may be associated with dearth of data on associated factors. Identifying these factors could inform the design and implementation of school-based programmes aimed at ameliorating these problems.

**Methods:**

A cross sectional survey involving 450 primary and secondary school children aged 6–15 years was conducted in Ede-Oballa, a rural community in Enugu state, South-eastern Nigeria. Selection of the pupils was by multistage sampling technique. Data were collected through interviewer administered questionnaire, anthropometric measurements of weight and height, 3-day weighed food intake, stool microscopy and blood analyses for malaria, zinc and vitamin A. Bivariate and multivariate logistic regression analyses were used to evaluate associations of interest with significance accepted at *P* < 0.05.

**Results:**

The school children were affected by underweight (18.2%), stunting (41.6%), thinness (20.0%), zinc (43.3%) and vitamin A (51.1%) deficiencies. After adjusting for potential confounders, weekly food expenditure was a major predictor of under-weight (AOR = 0.19, 95% CI: 0.08, 0.46), stunting (AOR = 0.36, 95% CI: 0.13, 0.95) and thinness (AOR = 0.49, 95% CI: 0.30, 0.80); household income was also a predictor of thinness (AOR = 0.47, 95% CI: 0.25, 0.88). Males had lower odds of being stunted than females (AOR = 0.31, 95% CI: 0.11, 0.83). The odd of being underweight was higher in female headed households than in households headed by males (AOR = 0.30, 95% CI: 0.12, 0.75). Tapeworm was an independent predictor of vitamin A (AOR = 3.59; 95% CI: 1.06, 12.13) and zinc (AOR = 3.64; 95% CI: 1.02, 12.98) deficiencies. Children with whipworm were more likely to be zinc (AOR = 3.80; 95% CI: 1.11, 13.04) and vitamin A (AOR = 3.79; 95% CI: 1.12, 12.89) deficient than those uninfected.

**Conclusion:**

Underweight, stunting, thinness, vitamin A and zinc deficiency among the school children were functions of weekly food expenditure, gender of household head and household income, tapeworm, whipworm, and sex. These findings emphasize the need for effective school- and community-based interventions.

## Background

Under-nutrition is a serious public health problem among children with huge consequences on the affected individuals, their families and the nation. Deficiencies of micronutrients such as iron, zinc and vitamin A affect a third to half of the world population [1] especially women, infants, and children in resource – poor households in low and medium income countries like Nigeria. Anthropometric deficits and micronutrient deficiencies have been shown [2–11] to be problems of public health significance among school children in different parts of Nigeria. A study conducted by Oninla, Owa, Onayade and Taiwo [2] on the nutritional status of children attending urban and rural public primary schools in Ife Central Local Government Area of Oyo State, Nigeria showed that children in both rural and urban areas were affected by anthropometric deficits with the rural school children being more disadvantaged. These nutrition problems are important constraints to effective child growth and development and compromise the school child’s ability to benefit maximally from his education. Poor nutrition and health among school children have been reported to contribute to the general inefficiency of education systems worldwide and improved nutrition and health lead to better performances, fewer repeated classes and reduced dropout rates [12]. School-aged children who suffered severe clinical under-nutrition in early childhood usually have poor cognition, school achievement, and motor skills, and have behaviour problems compared with matched controls [13].

Not much is being done to ensure good health and nutritional status of the school-aged child, particularly in the study area. This probably is a consequence of dearth of data on the factors associated with under-nutrition among school children. The school-aged child who is a survivor in an environment of high under-five morbidity and mortality is often not regarded as vulnerable and therefore not targeted for many nutrition and health programmes. Absence of functional school health programme further increases the vulnerability of these children to various nutrition and health problems that jeopardize their health and educational potentials.

Multiple factors may be responsible for the various forms of under-nutrition that affect Nigerian school children. Inadequate nutrient intake, infections (especially febrile and parasitic infections), and various socio-economic and environmental variables often lead to nutrition problems in children. Establishment of these factors is a major way of enhancing planning and implementation of interventions to curb the menace of these problems.

Although some studies on nutritional status of school children have been conducted in different parts of Nigeria, few have investigated the contributory factors of under-nutrition among school children and none to the best of our knowledge has been conducted in Enugu state. Enugu state is one of the states in which the Federal Government of Nigeria intended to commence school feeding. The data from this study would therefore inform policy makers on relevant factors to consider while planning school meal programme in the state and Nigeria as a whole. It was in this light that this study was undertaken to assess the prevalence of underweight, stunting, thinness, vitamin A and zinc deficiencies among rural school children aged 6–15 years in a South-eastern Nigerian community - Ede-Oballa and determine the factors associated with them.

## Methods

### Study design

The study employed a cross sectional survey design to determine the prevalence and predictors of under-nutrition among school children in Ede-Oballa.

### Study setting

Ede-Oballa is a typical rural community in South-eastern Nigeria made up of two large autonomous communities (Ede-Ukwu and Ede-Enu). It is located south of Nsukka town, the head quarters of Nsukka LGA of Enugu state. It had seven primary and three secondary schools, each densely populated. These were both state and private schools.

### Participants

The study comprised all free living (apparently healthy) primary and secondary school children aged 6–15 years in all ten schools in the study area. The study excluded sick children and those whose parents denied consent.

### Sample size and sampling technique

Sample size was derived from a single population proportion formula (4P (1 – P)/W^2^). We used vitamin A deficiency prevalence of 49.8% at 95% confidence interval and added 0.5% non response rate. The figure obtained was rounded off to 450 to reduce sampling error. The respondents were selected using multistage sampling technique: (a) identification of school children of 6–15 years in all the schools using school registers; (b) determination of the sample size per school and class by proportionate stratified sampling (with respect to age and sex); this was to ensure a wider representation and (c) the respondents were selected from each strata per school by simple random sampling technique. A subsample of 90 (20%) was randomly selected for weighed food intake and biochemical tests.

### Data collection methods

#### Questionnaire

General characteristics of the school children were elicited by the use of a validated and pretested questionnaire which was interviewer administered to each parent-child pair.

#### Anthropometry

A Harson’s bathroom salter scale (120 Kg capacity) was used in weight measurement. The scale was constantly maintained at zero prior to weighing each child. Each child was made to stand on the platform of the scale without shoes, hands by the sides and head held erect. Minimal clothing was allowed and reading was taken to the nearest 0.1 kg. Microtoise height metre rule was used to obtain the heights of the children. The children were made to stand erect without foot wears; with feet parallel, heels together, arms hanging by the sides and the buttocks, shoulder and back of the head touching the metre rule, the head piece was lowered gently to come in direct contact with the head. Readings were taken to the nearest 0.1 cm. Body mass index was calculated as the relationship of weight in kg to height in metres squared. Weight, height and BMI values were related to age and compared with WHO [14] child growth standards for weight-for-age, height-for-age and BMI-for-age z-scores. Children were classified as underweight, stunted and thin if z-scores for weight-for-age, height-for-age and BMI-for-age were less than or equal to -2SD below the WHO median of standards for age and sex. Overweight and obesity were taken as z-scores of +2SD and + 3SD, respectively.

#### Dietary assessment

Dietary assessment was carried out by a 3-day weighed food intake (2 weekdays and one weekend day) to determine energy, carbohydrate, and vitamin A and zinc intakes of 90 school children. All raw ingredients including the cook pot were weighed with kitchen scales prior to cooking and their values recorded. After cooking, the pot containing the food was weighed and the weight of the empty pot subtracted from it to obtain the weight of the cooked food. The quantity consumed by each child was obtained by subtracting plate wastes and leftovers from the portion given to the child. Snacks and foods bought and eaten outside the homes were estimated using household measures and the values recorded. Nutrients in the foods were calculated with the aid of food composition tables. The mean nutrient intake of the 3 days was related to recommended nutrient intakes (RNI) for these nutrients to obtain the percentage contributions. Adequate intake was taken as values equal to or above the RNI [15]. Data were not collected on feast/festival days as food intakes on these days may not reflect the usual food intake of the children.

#### Biochemical analyses

Serum vitamin A was assayed by High Performance Liquid Chromatography and deficiency was defined as serum retinol level of ≤20 μg/dl [16]. Serum zinc was determined by Atomic Absorption Spectrophotometry (AAS). Deficiency was defined as values less than 80 μg/dl [16]. Thin blood film method was used to test for malaria parasites in the erythrocytes of fresh whole blood. The wet mount direct method was used to analyze fresh stool for intestinal parasites.

#### Statistical analysis

Data were analyzed using the Statistical Package for Social Sciences (SPSS) for windows version 16.0. Descriptive statistics was used for general characteristics of the children and the results were presented in frequencies and percentages. Bivariate analysis (with Chi-square test) was employed to identify association between each of the independent variables and the response variables. Since chi-square analysis does not take into consideration confounding effects; we used multivariate logistic regression analysis (with stepwise backward elimination procedure) to adjust for simultaneous effects of multiple factors and to control the effects of confounding variables on the response variable. Odds ratios with 95% confidence interval were generated from this and used to determine the independent strength of the associations. *P* < 0.05 was considered significant.

## Results

### General characteristics of the respondents

The response rate observed in this study was 100%. Majority (63.1%) of the children had mothers who were aged 35 years and above. More than half of the mothers (67.3%) had less than secondary education. Majority were married (93.3%) and employed (89.6%). Few came from polygamous (20.2%) and single parent (18.0%) families. Female household heads constituted 45.3% while 70.0% had household monthly income of 25,000 Naira (79.43 US Dollar) and below. About 45% spent 5000 Naira (15.89 US Dollar) and below on food weekly. Energy, carbohydrate and vitamin A intakes were inadequate providing less than 100% of the RNI for 67.8%, 16.7% and 26.7% of the children, respectively. Zinc intake was adequate for all the children. Intakes were mainly plant based (Table 1).

**Table 1:** General characteristics of the school children (*N* = 450)

Variables	Frequency	Percentage	95% CI
Age of the mother			
15–34 years	166	36.9	30.0–51.1
35 years and above	284	63.1	48.9–70.0
Total	450	100.0	
Mother’s educational level			
Less than secondary education	303	67.3	23.3–43.3
Secondary education and above	147	32.7	56.7–76.7
Total	450	100.0	
Mother married			
Yes	420	93.3	11.1–27.8
No	30	6.7	72.2–98.9
Total	450	100.0	
Occupation			
Employed	403	89.6	58.2–97.1
Unemployed	47	10.4	7.6–13.3
Total	450	100.0	
Family type			
Monogamy	278	61.8	57.3–66.2
Polygamy	91	20.2	16.7–23.8
Single	81	18.0	14.7–21.8
Total	450	100.0	
Household head			
Male	246	54.7	50.0–59.1
Female	204	45.3	40.9–50.0
Total	450	100.0	
Household income			
25,000 Naira and below (≤79.43$)	315	70.0	61.8–78.9
Above 25,000 Naira (> 79.43$)	135	30.0	23.6–36.5
Total	450	100.0	
Weekly food expenditure			
5000 Naira and below (≤15.89$)	204	45.3	41.1–50.4
Above 5000 Naira (> 15.89$)	246	54.7	46.5–62.5
Total	450	100.0	
Energy intake (*N* = 90)			
Adequate (≥100% of RNI)	29	32.2	25.8–40.2
Inadequate (< 100% of RNI)	61	67.8	58.0–72.4
Total	90	100.0	
Carbohydrate intake (*N* = 90)			
Adequate (≥100% of RNI)	75	83.3	75.6–91.1
Inadequate (< 100% of RNI)	15	16.7	8.9–24.4
Total	90	100.0	
Vitamin A intake (*N* = 90)			
Adequate (≥100% of RNI)	66	73.3	64.4–83.3
Inadequate (< 100% of RNI)	24	26.7	16.7–35.6
Total	90	100.0	
Zinc intake (*N* = 90)			
Adequate (≥100% of RNI)	90	100.0	
Inadequate (< 100% of RNI)	0	0.0	
Total	90	100.0	

*RNI,* Recommended nutrient intakes, *CI Confidence interval*

### Prevalence of under-nutrition among the school children

Underweight was found among 18.2% of the school children; 20.0% of them were thin and 41.6% were stunted. Only 9.3% were overweight and no (0%) child was obese. Zinc deficiency affected 43.3% and 51.1% were vitamin A deficient. School children who were aged 6–9 years had the highest (56.2%) prevalence of vitamin A deficiency while the highest prevalence of stunting (45.9%) and thinness (28.6%) was found among the 13–15 year olds. The 10-12 year olds had the highest prevalence of underweight (31.1%) and zinc deficiency (48.0%). On chi square analysis, the prevalence of underweight, stunting and thinness increased significantly with age. The relationship was stronger with thinness (*P* < 0.001) and stunting (*P* < 0.01) (Table 2).

**Table 2:** Anthropometric indices, serum vitamin A and zinc status of the school children

	6–9 years	10–12 years	13–15 years	Total		
Variables	N (%)	N (%)	N (%)	N (%)	*P value	95% CI
Weight-for-age (*N* = 176)						
Underweight	23(15.6)	9(31.1)	…….	32(18.2)	0.037	10.3–27.3
Normal	109(74.2)	20(68.9)	….....	129(73.3)		59.1–87.5
Overweight	15(10.2)	0(0.0)	…….	15(8.5)		4.5–13.1
Total	147(100.0)	29(100.0)	…….	176(100.0)		
Height-for-age (*N* = 450)						
Stunted	50(34.0)	52(44.1)	85(45.9)	187(41.6)	0.002	25.0–45.5
Normal	97(65.0)	66(55.9)	100(54.1)	262(58.4)		47.7–75.6
Total	147(100.0)	118(100.0)	185(100.0	450(100.0)		
Body Mass Index-for-age (*N* = 450)						
Thinness	20(13.6)	17(14.4)	53(28.6)	90(20.0)	0.000	6.8–22.1
Normal	94(70.7)	91(77.1)	123(66.5)	318(70.7)		58.5–88.1
Overweight	23(15.7)	10(8.5)	9(4.9)	42(9.3)		8.0–18.2
Total	147(100.0)	118(100.0)	185(100.0)	450(100.0)		
Serum vitamin A status (*N* = 90)						
Deficient (≤20 μg/dl)	18(56.2)	11(44.0)	17(51.5)	46(51.1)	0.497	41.1–61.1
Normal (> 20 μg/dl)	14(43.8)	14(56.0)	16(48.5)	44(48.9)		38.9–58.9
Total	32(100.0)	25(100.0)	33(100.0)	90(100.0)		
Serum zinc status (*N* = 90)						
Normal (≥80 μg/dl)	17(53.1)	13(52.0)	21(63.6)	51(56.7)	0.595	46.7–66.7
Deficient (< 80 μg/dl)	15(46.9)	12(48.0)	12(36.4)	39(43.3)		33.3–53.3
Total	32(100.0)	25(100.0)	33(100.0)	90(100.0)		

*P values were generated through chi square analysis, *CI Confidence interval*

### Predictors of underweight, stunting, thinness, zinc and vitamin A deficiencies

Results of bivariate analysis of factors associated with underweight, stunting and thinness among the school children were presented in Table 3. Gender of household head (COR = 0.30, 95% CI: 0.13, 0.70), household income (COR = 0.31, 95% CI: 0.11, 0.85), weekly food expenditure (COR = 0.18, 95% CI: 0.08, 0.40) and age of the child (COR = 2.43, 95% CI: 0.98, 5.99) were significant relative risk factors associated with underweight. Significant risk factors for stunting were household income (COR = 0.61, 95% CI: 0.40, 0.93), weekly food expenditure (COR = 0.55, 95% CI: 0.38, 0.80), sex of the child (COR = 0.68, 95% CI: 0.47, 0.99), age of the child (COR = 1.60, 95% CI: 1.06, 2.41), malaria (COR = 0.34, 95% CI: 0.14, 0.81) and tapeworm (COR = 0.38, 95% CI: 0.15, 0.99). Gender of household head (COR = 0.60, 95% CI: 0.37, 0.97), household income (COR = 0.36, 95% CI: 0.19, 0.67), weekly food expenditure (COR = 0.40, 95% CI: 0.25, 0.64), age of the child (COR = 1.91, 95% CI: 1.11, 3.28), and school level (COR = 1.60, 95% CI: 1.01, 2.55) were risk factors that had significant associations with thinness.

**Table 3:** Bivariate analysis of factors associated with underweight, stunting and thinness among the school children

Variables	Weight - for - age	Height - for - age	BMI – for - age
	COR (95% CI)	COR (95% CI)	COR (95% CI)
*Characteristics of the school children*			
Female school children	1.67(0.76–3.66)	0.68(0.47–0.99)*	1.03(0.65–1.64)
Older school children (10–15 years)	2.43(0.98–5.99)*	1.60(1.06–2.41)*	1.91(1.11–3.28)*
Secondary school children		1.07(0.73–1.56)	1.60(1.01–2.55)*
*Mothers’/Household characteristics*			
35 years and above	0.55(0.25–1.19)	1.00(0.68–1.47)	0.95(0.59–1.54)
≥ Secondary education	1.39(0.64–2.99)	1.00(0.67–1.49)	0.70(0.42–1.17)
Female household head	0.30(0.13–0.70)**	0.90(0.62–1.32)	0.60(0.37–0.97)*
Income above 25,000 Naira (> 79.43$)	0.31(0.11–0.85)*	0.61(0.40–0.93)*	0.36(0.19–0.67)**
Weekly food expenditure > 5000 Naira (> 15.89$)	0.18(0.08–0.40)***	0.55(0.38–0.80)**	0.40(0.25–0.64)***
*Intakes*			
Inadequate energy intake	0.38(0.08–1.82)	1.06(0.43–2.64)	0.66(0.25–1.78)
Inadequate carbohydrate intake		2.03(0.66–6.22)	2.28(0.71–7.29)
*Micronutrient status*			
Vitamin A deficiency	1.53(0.32–7.29)	1.23(0.53–2.88)	1.34(0.52–3.48)
Zinc deficiency	3.00(0.63–14.37)	1.42(0.60–3.33)	1.28(0.49–3.31)
*Parasitic infections*			
Malaria present	0.24(0.05–1.16)	0.34(0.14–0.81)*	1.12(0.42–2.94)
Hookworm present	0.57(0.10–3.27)	0.69(0.28–1.68)	0.89(0.33–2.41)
Roundworm present	2.00(0.38–10.41)	1.34(0.53–3.43)	0.89(0.31–2.60)
Tapeworm present	1.88(0.42–8.44)	0.38(0.15–0.99)*	1.23(0.46–3.27)
Whipworm present	0.49(0.09–2.81)	0.52–0.21-1.32)	1.69(0.64–4.46)
*Entamoeba hystolitica* present	1.25(0.28–5.59)	0.71(0.30–1.69)	0.39(0.14–1.10)

*COR* Crude odds ratio, *CI* Confidence interval, *BMI* Body mass index*,* *P < 0.05 **P < 0.01 ***P < 0.001

After controlling for all other independent variables, gender of household head was found to be an independent predictor of underweight (AOR = 0.30, 95% CI: 0.12, 0.75). Weekly food expenditure proved a significant determinant of underweight (AOR = 0.19, 95% CI: 0.08, 0.46), stunting (AOR = 0.36, 95% CI: 0.13, 0.95) and thinness (AOR = 0.49, 95% CI: 0.30, 0.80). The odds of being thin were lower among children from low income households (AOR = 0.47, 95% CI: 0.25, 0.88). Sex was also an important predictor of stunting (Table 4). The odds of being stunted were higher among female children (AOR = 0.31, 95% CI: 0.11, 0.83).

**Table 4:** Multivariate analysis of risk factors for underweight, stunting and thinness among the school children

Independent variables	AOR	95% CI	*P* value
	Underweight		
Female household head	0.30	0.12–0.75	0.010*
Income above 25,000 Naira (79.43$)	0.63	0.21–1.92	0.419
Weekly food expenditure above 5000 Naira (15.89$)	0.19	0.08–0.46	0.000***
Older school children (10–15 years)	1.57	0.56–4.44	0.392
Female school children	1.51	0.63–3.58	0.354
	Stunting		
Income above 25,000 Naira (79.43$)	1.01	0.31–3.29	0.986
Weekly food expenditure above 5000 Naira (15.89$)	0.36	0.13–0.95	0.039*
Female school children	0.31	0.11–0.83	0.020*
Older school children (10–15 years)	1.45	0.51–4.16	0.488
Malaria present	0.37	0.12–1.10	0.072
Tapeworm present	0.69	0.22–2.20	0.530
	Thinness		
Secondary school children	1.20	0.66–2.16	0.550
Income above 25,000 Naira (79.43$)	0.47	0.25–0.88	0.019*
Weekly food expenditure above 5000 Naira (15.89$)	0.49	0.30–0.80	0.005**
Older school children (10–15 years)	1.35	0.68–2.69	0.392
Female school children	0.68	0.42–1.12	0.129

*AOR* Adjusted odds ratio, *CI* Confidence interval, *P < 0.05 **P < 0.01 ***P < 0.001

Bivariate analysis of factors associated with vitamin A deficiency (VAD) showed that tapeworm (COR = 3.89, 95% CI: 1.53, 9.89) and whipworm (COR = 3.57, 95% CI: 1.40, 9.07) increased the risk of having VAD. After simultaneous adjustment for all confounders, multivariate logistic regression analysis showed that the association between tapeworm (AOR = 3.59, 95% CI: 1.06, 12.13) and whipworm (AOR = 3.79; 95% CI: 1.12, 12.89) with VAD remained significantly (*P* < 0.05) strong (Table 5). The odds of having zinc deficiency was significantly lower among children who were infected with tapeworm (AOR = 3.64, 95% CI: 1.02, 12.98) (P < 0.05) and whipworm (AOR = 3.80, 95% CI: 1.11, 13.04) (P < 0.05); and those who had males as their household heads (AOR = 0.23, 95% CI: 0.07, 0.69) (*P* < 0.01). The relationship between intakes and under-nutrition was not significant (*P* > 0.05).

**Table 5:** Predictors of vitamin A and zinc deficiencies among the school children

Variables	Serum vitamin A		Serum zinc	
	COR (95% CI)	AOR (95% CI)	COR (95% CI)	AOR (95% CI)
*Characteristics of the school children*				
Female school children	1.70 (0.74–3.94)	1.47(0.55–3.94)	1.15 (0.50–2.66)	1.15(0.42–3.17)
Older school children (10–15 years)	0.73 (0.31–1.73)	1.15(0.37–3.57)	0.80 (0.34–1.91)	1.38(0.44–4.36)
*Mothers’/Household characteristics*				
35 years and above	0.51 (0.22–1.20)	0.45(0.16–1.29)	0.43 (0.18–1.03)	0.35(0.12–1.07)
≥ Secondary education	1.14 (0.48–2.75)	0.79(0.27–2.37)	1.00 (0.41–2.42)	0.49(0.15–1.63)
Female household head	1.83(0.77–4.38)	1.43(0.51–4.01)	2.38 (0.96–5.89)	0.23(0.07–0.69)**
Income > 25,000 Naira (79.43$)	1.70 (0.65–4.47)	2.97(0.79–11.16)	1.01(0.39–2.62)	2.28(0.62–8.37)
WFE > 5000 Naira (15.89$)	0.99 (0.44–2.28)	0.93(0.35–2.49)	1.12 (0.49–2.59)	1.09(0.41–2.94)
*Nutrient intake*				
Inadequate vitamin A intake	0.94 (0.37–2.39)	0.72(0.21–2.48)		
*Parasitic infections*				
Malaria present	1.71 (0.73–3.99)	0.36(0.09–1.44)	2.16 (0.90–5.18)	0.65(0.17–2.56)
Hookworm present	1.51 (0.64–3.58)	1.06(0.35–3.24)	1.15 (0.48–2.72)	0.58(0.18–1.88)
Round worm present	1.05 (0.42 (2.64)	0.94(0.29–2.99)	1.63 (0.64–4.11)	2.40(0.71–8.12)
Tapeworm present	3.89 (1.53–9.89)	3.59(1.06–12.13)*	3.42 (1.39–8.43)	3.64(1.02–12.98)*
Whipworm present	3.57 (1.40–9.07)	3.79(1.12–12.89)*	3.83 (1.53–9.57)	3.80(1.11–13.04)*
*Entamoeba hystolitica* present	1.11 (0.48–2.57)	1.07(0.37–3.06)	1.93(0.82–4.52)	1.01(0.34–3.02)

*COR* Crude odds ratio, *AOR* Adjusted odds ratio, *CI* Confidence interval, *WFE* Weekly food expenditure *P < 0.05 **P < 0.01

## Discussion

Underweight, stunting, thinness, zinc and vitamin A deficiencies were prevalent among the school children. The prevalence of stunting, underweight and thinness reported in this study was higher than the values reported by Nwamara et al. [6] but lower than the report of Atawodi et al. [3]. Vitamin A deficiency prevalence in this study was higher than the findings of Atimati et al. [10] and Egbi [17]. Both reported vitamin A deficiency prevalence of 29.6% and 35.6%, respectively. Abah et al. [8] reported a much higher prevalence (99.2%) of zinc deficiency than observed in this study. The consequences of these nutrition problems are often staggering with high morbidity due to lowered immunity and compromised physiology and may be associated with mortality among affected school children. The high morbidity may result to high absenteeism from school as Mahawithanage et al. [18] affirmed that better serum vitamin A status is associated with improved school attendance. We observed that these nutrition problems were associated with multiple factors.

According to this study, the prevalence of underweight, stunting and thinness, vitamin A and zinc deficiencies was lower among children whose household heads were females. This was significant (*P* < 0.05) for underweight and zinc deficiency only. This finding was not a surprise. Culturally, women are better care takers of children than men. Some have been observed to go hungry to ensure that food was enough for their children especially in households that are food insecure. Ene-Obong et al. [19] affirmed that most women gave their children and husbands preference in food distribution. Johnson and Rogers [20] noted that children from female headed households are at least well nourished compared to those living in male headed households of the same income class. This emphasizes the need to empower women to ensure higher socioeconomic power and therefore better nutritional status of their children. In support of this, Donkoh et al. [21] showed that more female headed households spent greater percentage of their incomes on food than male headed households. Culturally, females become household heads due to physical absence of their spouses as a result of migration, divorce/separation, abandonment and death.

Weekly food expenditure was shown in this study to be an important predictor of underweight, stunting and thinness. Ochieng’ [22] and Torlesse et al. [23] reported similar findings. This result was expected because when income is low, the amount of money spent on food will invariably be low implying higher food insecurity in low income households especially when most foods are not in season. Household income has been identified by other authors [24, 25] as a factor causing nutrition problems in children. Most often when income is limited, food and food related expenditures in households are the most compromised. Higher food expenditure may imply increased access to food, higher diversity scores and lower prevalence of under-nutrition because improvement in both quality and quantity of food consumed is guaranteed.

Sex was found to be an independent determinant of stunting among the study group. The higher prevalence among males is in agreement with observations by other researchers [26–28]. Kabubo-Mariara et al. [28] reported that boys are more likely than girls to suffer from chronic and acute under-nutrition. Mwaniki and Makokha [29] reported that boys aged 4–7 and 8–11 years had a higher risk of being stunted as compared to girls of the same age. This may be a result of inadequate nutrient intake and higher physical activity levels. Lenhart et al. [30] reported that girls were less likely to be active than boys: 27.9% of girls were sedentary as compared to 10.6% of boys. Besides, males have higher basal metabolic rates. These may lead to higher energy expenditure among them and in the face of inadequate intake may lead to higher prevalence of under-nutrition especially stunting, an index of chronic nutrition deprivation.

In this study, we found that tapeworm and whipworm infections were significantly associated with vitamin A and zinc deficiencies. These are endoparasites which depend on their hosts for foods thus depriving the host of essential nutrients. Quihui-Cota et al. [31] reported higher z-scores for weight-for-height, weight-for-age, and height-for-age among school children uninfected with intestinal helminths and showed that higher prevalence of intestinal infections in children was associated with lower height-for-age and weight-for-age z-scores. Negative relationship between helminths and nutrition status of children has also been reported by other authors [32–34].

Zinc concentration was also reported to be lower in children with soil transmitted helminths [35]. We opined that both tapeworm and whipworm may have had heavier intestinal load than other intestinal parasites. It probably could also be that these two helminths had longer infective periods than other parasites. Wolde et al. [12] showed a significant relationship between trichuris and stunting; but not with underweight and wasting and asserted that this was a result of chronic nature of trichuris infection. We found no significant association with underweight, stunting and thinness.

Though not significant, we observed that the prevalence of underweight, stunting and thinness was higher among children who had inadequate energy intake. Mwaniki and Makokha [29] reported similar findings. They reported that children who had adequate energy intake were less likely to be underweight than those who had inadequate energy intake. We observed that nutrient intakes were based mainly on plant foods which have low bioavailability. This may be responsible for the lack of significant association between nutrient intake and nutrition status despite intake that exceeded 100% of the recommended intakes. Though zinc intake was adequate for all the children, we observed zinc deficiency of severe public health importance implying that intake was not reflected on serum zinc status of the children. This calls for bioavailability assessment of our commonly consumed foods.

## Limitation of the study

The study was conducted in a rural community. Though large with ten schools, it is not a true representation of the entire rural areas in Nigeria. However, it has affirmed that nutrition problems among rural school children are public health problems that require a focused multisectoral school- and community-based approaches for their control.

## Conclusion

Among school children in Ede-Oballa, underweight, stunting, thinness, vitamin A and zinc deficiencies were nutrition problems of public health significance. Weekly food expenditure was an independent predictor of underweight, stunting and thinness. Gender of household head was also a predictor of underweight. Sex was an independent predictor of stunting and household income was significantly associated with thinness. Gender of household head, whipworm and tapeworm were major factors contributing to zinc deficiency. Tapeworm and whipworm were also important determinants of vitamin A deficiency. Empowerment of females through free education for the girl-child, and loans to adult females is necessary to ensure sustained better nutritional status of children. A functional school health and feeding programme is vital to improve nutrient intake and enhance deworming and vitamin A supplementation among school children to boost their immunity and ensure better nutritional and health status.
